# Neutralization sites of human papillomavirus-6 relate to virus attachment and entry phase in viral infection

**DOI:** 10.1080/22221751.2019.1694396

**Published:** 2019-11-26

**Authors:** Xinlin Liu, Jie Chen, Zhiping Wang, Daning Wang, Maozhou He, Ciying Qian, Shuo Song, Xin Chi, Zhibo Kong, Qingbing Zheng, Yingbin Wang, Hai Yu, Qinjian Zhao, Jun Zhang, Shaowei Li, Ying Gu, Ningshao Xia

**Affiliations:** a State Key Laboratory of Molecular Vaccinology and Molecular Diagnostics, School of Life Sciences, Xiamen University, Xiamen, People’s Republic of China; b National Institute of Diagnostics and Vaccine Development in Infectious Disease, School of Public Health, Xiamen University, Xiamen, People’s Republic of China

**Keywords:** Human papillomavirus type 6, antibody, viral neutralization, cryo-EM structure, neutralization mechanism

## Abstract

Human papillomavirus type 6 (HPV6) is the major etiologic agent of genital warts and recurrent respiratory papillomatosis. Although the commercial HPV vaccines cover HPV6, the neutralization sites and mode for HPV6 are poorly understood. Here, we identify the HPV6 neutralization sites and discriminate the inhibition of virus attachment and entry by three potent neutralizing antibodies (nAbs), 5D3, 17D5, and 15F7. Mutagenesis assays showed that these nAbs predominantly target surface loops BC, DE, and FG of HPV6 L1. Cryo-EM structures of the HPV6 pseudovirus (PsV) and its immune complexes revealed three distinct binding modalities – full-occupation-bound to capsid, top-center-bound-, and top-rim-bound to pentamers – and illustrated a structural atlas for three classes of antibody-bound footprints that are located at center-distal ring, center, and center-proximal ring of pentamer surface for 5D3, 17D5, and 15F7, respectively. Two modes of neutralization were identified: mAb 5D3 and 17D5 block HPV PsV from attaching to the extracellular matrix (ECM) and the cell surface, whereas 15F7 allows PsV attachment but prohibits PsV from entering the cell. These findings highlight three neutralization sites of HPV6 L1 and outline two antibody-mediated neutralization mechanisms against HPV6, which will be relevant for HPV virology and antiviral inhibitor design.

**Highlights**
- Major neutralization sites of HPV6 were mapped on the pseudovirus cryo-EM structure
- mAb 15F7 binds HPV6 capsid with a novel top-rim binding modality and confers a post-attachment neutralization
- mAb 17D5 binds capsid in top-centre manner but unexpectedly prevents virus from attachment to cell surface

## Introduction

Human papillomavirus (HPV) infection is a leading cause of genital warts and cervical cancer [1]. More than 200 genotypes of HPV have been identified based on the homology of the major capsid protein, L1 [2]. According to its propensity to cause cervical cancer, HPV is classified into low- or high-risk types. HPV6 and HPV11 are two major types of low-risk HPV and are responsible for over 90% of genital warts [3–9]. There are three commercially available HPV prophylactic vaccines: Cervarix, Gardasil 4, and Gardasil 9; the latter two contain HPV6 and 11 antigens, which can provide protection against HPV6 and 11 infection with high efficacy [10, 11], however, these vaccines offer little therapeutic benefit against existing infection.

The current vaccines on the market were designed based on the mode of self-assembly of L1 to form virus-like particles (VLPs). L1 can interact with cellular receptors, such as heparan sulfate proteoglycans (HSPGs) or laminin-332 on the cell surface to drive primary viral attachment to host cells [12]. The HPV minor structural capsid protein, L2, on the other hand, is involved with cell entry and viral genome escape [13]. The L1 protein alone or together with L2 can assemble into VLPs [14], and induce a strong immune response from the host against infection [15]. The 55∼60-nm diameter HPV capsid is composed of 72 L1 protein pentamers, each consisting of five copies of L1 [16], and an uncertain number of L2 proteins. Twelve of the pentamers lie on the icosahedral 5-fold axes (pentavalent capsomers), while the other 60 pentamers are positioned at the pseudo 6-fold axes (hexavalent capsomers) [17]. Crystal structures of L1 pentamers (HPV11, -16, -18, and -35) have illustrated that the HPV L1 monomer forms a canonical, eight-stranded-barrel (BIDG-CHEF) linked by six highly variable loops (BC, CD, DE, EF, FG, and HI)[18]. These surface loops, except CD, contain the highest sequence variation among the different HPV types and play a critical role in eliciting the responses of protective antibodies by the host. As such, most of the neutralizing epitopes have been identified at these hypervariable loops, with various sites recognized by type-specific neutralizing antibodies (nAbs) [17, 19, 20].

To establish infection, HPV undergoes a complicated process that includes receptor binding, a post-attachment conformational change, furin cleavage, and internalization [21]. HSPGs, located on the basement membrane in the tissues, are the primary receptors for HPV infection, and facilitate upstream events, including the initial attachment and conformational changes of the capsid protein [22, 23]. Co-crystallization of HPV16 with heparin revealed four distinct heparin binding sites that were critical for the conformational changes that permit virus access and mediate downstream events, including secondary binding site exposure, transfer to the uptake receptor, and capsid uncoating [23]. In addition to HSPGs, several other cellular receptors have been identified, such as α6 integrin, growth factor receptors, cyclophilin B (CyPB), laminin-332 and annexin A2 [24, 25]. However, the specific details of these interactions remain unclear.

Virus neutralization is defined as an abrogation of virus infection through antibody-virus associations. Previous studies have highlighted the different neutralization mechanisms mediated by HPV nAbs [26]. Antibodies such as HPV16.V5 and HPV16.E70 do not prevent virus from binding to the primary cellular attachment receptor (HSPG), but are proposed to block the subsequent shift to engage other co-receptors [23]. Other Abs, such as HPV16.U4, may interfere with the virus-HSPG/receptor interaction or the ensuing endocytic process [26, 27]. Cryo-EM structures of the HPV capsid in complex with nAbs indicate that, for most nAbs, the core binding epitopes involve distinct surface loops of the L1 protein: FG loop (HPV16.V5, HPV16.E70, HPV59.28F10), HI loop (HPV16.V5, HPV16.E70), and DE loop (HPV58.A12A3). In contrast, HPV16.U4 uniquely recognizes the C-terminal arm of L1. Amino acid residues 49–54 and 169–178 of HPV6 L1 are the principal regions for HPV6 type-specific antibody recognition, as identified via studies with the cottontail rabbit papillomavirus (CRPV)/HPV6-hybrid L1 protein [28]. Yet, and in part due to a lack of high-resolution structural information, the exact epitopes involved in HPV6 neutralization remains unknown, as does the molecular mechanism of the interaction.

In this study, we tested three potent neutralizing antibodies against HPV6 using epitope mapping and structural analysis. Through homologous loop-swapping of the L1 protein between HPV6 and HPV16, we identified the BC, DE, and FG loops as the key surface loops responsible for type-specific antibody recognition. To verify the variability in binding, we constructed cryo-EM structures of HPV6 PsV complexed with Fabs and unveiled the epitope locations targeted by these nAbs. Using immunofluorescence, we identified three patterns of binding and overall two modes of neutralization. Overall, these findings offer insight into the mechanism(s) of low-risk HPV neutralization mediated by antibodies.

## Materials and Methods

### Cell lines

HaCaT cells and 293FT cells were cultivated in Dulbecco’s modified Eagle’s medium supplemented with 10% fetal bovine serum (FBS). These cells were used for HPV6 PsV production (293FT cells), immunofluorescence microscopy (HaCaT cells), and the PsV neutralization assay (293FT cells).

### Monoclonal antibodies (mAbs)

BALB/c mice for hybridoma preparation were immunized subcutaneously three times with HPV6 VLPs absorbed with aluminum adjuvant as described in our previous study [29]. A set of anti-HPV6 murine mAbs were generated by ascites fluid in a standard hybridoma assay and screened by enzyme-linked immunosorbent assay and pseudovirus-based neutralization assay. These antibodies were affinity-purified on Protein A columns. The concentrations of the purified murine IgGs were determined at OD_280nm_ before dilution in PBS and storage in a final concentration of 1.0 mg/ml at −20°C.

### Preparation of the HPV6 PsV

HPV6 PsVs were produced as described in our previous study [29]. The L1/L2 expression vector p6sheLLr (No. 37318) was obtained from Addgene and reporter plasmid pN31-EGFP was kindly provided by Dr.J.T.Schiller [30]. The plasmids encoding HPV6 L1 and L2 proteins and the reporter plasmid were co-transfected into 293FT cells. 293FT cells were harvested 72 h after transfection, lysed in lysis buffer containing 0.5% Brij58 (Sigma-Aldrich), 0.2% Benzonase (Merck Millipore, Darmstadt, Germany), 0.2% PlasmidSafe ATP-Dependent DNase (Epicenter Biotechnologies, Madison, WI), and Dulbecco’s phosphate-buffered saline (DPBS)-Mg solution, and then incubated at 37°C for 18 h. Lysates were then combined with 5 M NaCl. PsV titers were determined using the tissue culture infective dose (TCID_50_) values, measured using the Reed-Muench method [31].

### Construction and purification of HPV6-16 chimeric VLPs

We replaced each of the surface loops of the HPV6 L1 protein with the homologous loops of the HPV16 L1 protein separately, hereafter referred to as HPV6-16 chimeric VLPs. Genes encoding the HPV16 loop (BC, DEa, DEb, DEc, EF, FGa, FGb, FGc, HIa, HIb) were cloned into pTO-T7-HPV6 L1 to replace HPV6 loops by Gibson Assembly [29]. The recombinant plasmids were transformed into *E.coli* ER2566 strain for the expression of the HPV6-16 loop mutant VLPs. The transformed cells were cultured in LB medium at 37°C overnight, and protein expression was induced by the addition of isopropyl-β-D-thiogalactoside at 25°C for 8 h. Bacterial cells were collected by centrifugation and re-suspended in cell lysis solution (20 mM Tris, pH 7.2, 300 mM NaCl, 10 mM EDTA). After sonication, the target proteins were released from cells and were separated by centrifugation. The lysate supernatant was combined with 20 mM DTT to denature the protein. Target proteins were purified using an SP Sepharose 4 Fast Flow column (GE Healthcare) and eluted with a solution containing 20 mM PB8.0, 20 mM DTT and 800 mM NaCl. Proteins were further purified using a CHT-II column (Bio-Rad), with elution in 20 mM PB8.0, 20 mM DTT and 1 M NaCl. Protein purity was assessed using sodium dodecyl sulfate-polyacrylamide gel electrophoresis (SDS-PAGE), according to the Laemmli method.

### Enzyme-linked immunosorbent assay (ELISA)

HPV6-16 chimeric VLPs and HPV6 wild-type VLPs were coated into the wells of a 96-well microplate at a concentration of 300 ng per well and then incubated with serial dilutions of each monoclonal antibody at 37°C for 45 min. The wells were washed, incubated with HRP-conjugated goat anti-mouse IgG at 37°C for 45 min, and then washed again. Tetramethylbenzidine substrate (100 μl) was added to each well and the plates incubated at 37°C for 10 min. The reaction was stopped with 2 M H_2_SO_4_, and the OD values were measured at 450 nm, with a reference wavelength of 620 nm. GraphPad Prism 7 was used to assess the median effective concentration (EC_50_) of each monoclonal antibody.

### Transmission electron microscopy (TEM)

The morphologies of the HPV6-16 chimeric VLPs diluted to 200 μg/ml were analyzed by negative staining TEM using an FEI Tecnai Spirit TEM at 120 kV and imaged at approximately 25,000× magnification. The approach was used to confirm the full-length antibodies capable to bind HPV6 VLPs as well, the samples were prepared by incubating the HPV6 VLP with excessive amount of full-length antibodies at 37°C for 2h.

### Pseudovirus-based neutralization assay (PBNA)

293FT cells were cultured in 96-well plates at a density of 1.5 × 10^4^ cells per well and incubated at 37°C for 4 h. Monoclonal antibody samples were diluted from 1,000–0.488 ng/mL with two-fold serial diluent (DMEM) and the PsVs were diluted to 2 × 10^5^ TCID_50_/μl. Equal volumes (60 μl) of PsV diluent and the serially diluted antibodies were mixed in each well, and incubated at 37°C for 1 h. 293FT cells were then incubated with 100 μl of the mixtures at 37°C for 72 h. The median inhibitory concentration (IC_50_) was defined as the antibody concentration for achieving 50% inhibition of PsV.

### Immunoflurescence microscopy

HaCaT cells were seeded onto coverslips in 24-well plates and incubated for 48 h. To study the binding model, the PsV (200 ng) was incubated with monoclonal antibodies at a neutralization dose at 37°C for 1 h and then added to the prepared HaCaT cells for 1 h at room temperature. Cells were then fixed in 4% paraformaldehyde in PBS. For the detection of antibody-bound particles, the cells were stained with Alexa Fluor 488-conjugated donkey anti-mouse IgG. For the detection of particles mixed with BSA, the cells were stained with a mouse polyclonal antiserum raised against HPV6 and subsequently stained with Alexa Fluor 488-conjugated donkey anti-mouse IgG. Rhodamine-conjugated phalloidin was used to delineate the cell body.

To evaluate the ECM-binding model, the ECM was firstly prepared as described below. Briefly, HaCaT cells were seeded onto the coverslips for 48 h and then were then removed by lysis buffer digestion (0.5% Triton X-100, 10 mM NH_3_·H_2_O, 1 unit/ml Dnase-I in PBS). The coverslips were washed three times with PBS, leaving the ECM on the coverslips. PsV pre-incubated with monoclonal antibody were added to the ECM, which was subsequently fixed in 4% paraformaldehyde in PBS. The antibody-bound particles were detected with Alexa Fluor 488-conjugated donkey anti-mouse IgG. Laminin 5 was detected with a rabbit polyclonal antiserum and Alexa Fluor 594-conjugated donkey anti-rabbit IgG. In all cases, the cells were counterstained with 4’,6-diamidino-2-phenylindole (DAPI). All immunofluorescence images were captured using a Zeiss LSM 780 confocal system. Images were processed with Adobe Photoshop software.

### Cryo-EM and three-dimensional (3D) reconstruction

To prepare the PsV-Fab immune complexes, the purified HPV6 PsV was mixed with an oversaturated amount of Fab and then incubated at 37°C for 2 h. The sample (2 μl) was pipetted onto a glow-discharged Quantifoil holey carbon grid (R2/1, 200 mesh; Quantifoil Micro Tools) in a FEI Vitrobot. For the HPV6:5D3 and HPV6:17D5 complexes, cryo-EM images were collected on a FEI Falcon 2 direct detector camera using a FEI TF30 FEG microscope operated at 300 kV at a 93,000× nominal magnification. The electron dose was determined at 25 e-/Å^2^. Robem was used to box and extract the particles [32]. Complex image processing and 3D reconstruction were completed using Relion and AUTO3DEM programs [32]. For the HPV6 particles and the HPV6:15F7 complex, a Falcon 3 direct detector camera and cisTEM were used to complete the above work. For a higher resolution structure of the HPV6 particle, we reconstructed subareas surrounding the 2-fold axes of the icosahedral HPV6 capsid using Relion and cisTEM.

### HPV6 model building and fitting of HPV6 particles and Fab

The cryo-EM structure of the HPV16 virus protomer (PDB ID:5KEP) was used for homology modeling and fitted by Chimera [33] into the corresponding volume of the subparticle density map. Coot [34] and Phenix [35] were used to modify and refine the atomic positions. Chimera was used for visualization and segmentation of the density maps [33]. The HPV6 particle model and the crystal structure of the Fab (PDB ID:3RKD) were fitted into the immune complex maps by the “fit in the map” command in UCSF Chimera. Fab density in the cryo-EM maps were projected on a stereographic sphere by RIVEM [36].

### Data bank accession numbers

Atomic coordinates of HPV6 particle have been submitted to the Protein Data Bank with accession number 6L31. The cryo-EM density maps for HPV6 subparticle, HPV6 particle alone and in complex with antibodies have been deposited in the Electron Microscopy DataBank (EMDB: EMDB-0816, EMDB-0817, EMDB-0818, EMDB-0819, EMDB-0820).

## Results

### Binding region profiles of potent HPV6 nAbs

**Figure 1.**
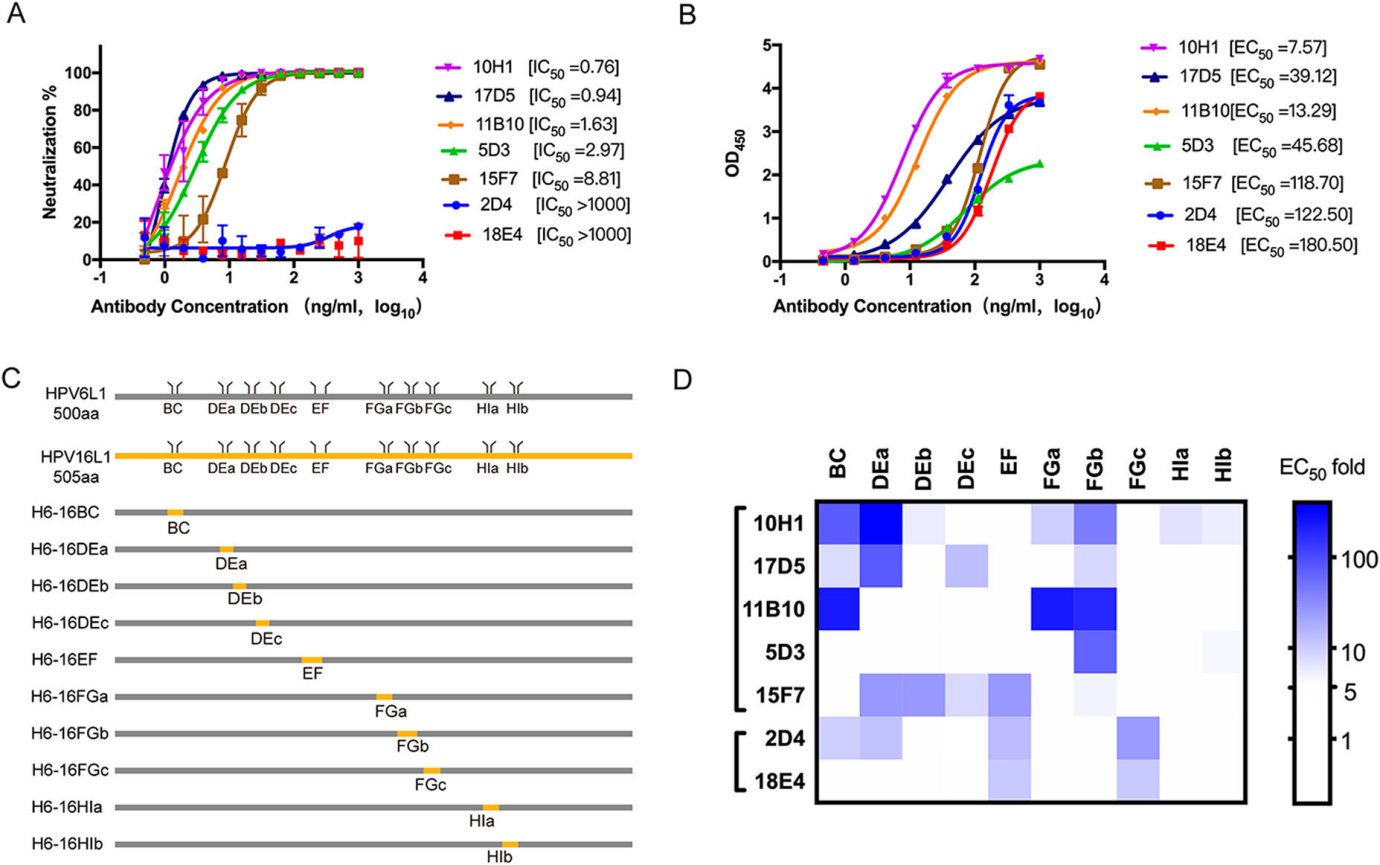
Characterization of a HPV6 mAb panel. (A) Neutralization efficiency, reflected as IC_50_, was determined through pseudovirus-based neutralization assay (PBNA). (B) Binding activity, reflected as EC_50_, was determined using an enzyme-linked immunosorbent assay (ELISA) using HPV6 VLPs. (C) Schematic representation of the constructs of the wild-type and chimeric HPV6-16 L1 proteins. Sequences of the five surface loops for each WT or mutated L1 protein are indicated. (D) Epitope analysis of seven mAbs displayed as a heat map. The colours ranging from white to blue denote the increase in EC_50_ value in the ELISA. Darker shades of blue indicate critical regions for mAb binding.

We first sought to investigate the epitope structural characteristics and neutralization mechanism of HPV6 nAbs, selecting the five most potent nAbs (10H1, 17D5, 11B10, 5D3, and 15F7) and two non-neutralizing antibodies (2D4 and 18E4) from a panel of HPV6 type-specific murine mAbs that was generated in our previous work [29]. As expected, the five neutralizing antibodies showed high neutralizing efficiency (IC_50 _< 10 ng/ml) against the HPV6 PsV in the PBNA assay. However, mAb 15F7 exhibited a slightly lower neutralization activity (8.81 ng/ml) than the other mAbs (Figure 1A); a similar difference for 15F7 was also noted in the binding efficiency against HPV6 VLP in the EC_50_ assay (Figure 1B). As expected, the non-neutralizing antibodies, 2D4 and 18E4, showed no neutralization activity.

The viral epitopes that induce the production of nAbs against HPV are mainly located on the surface loops of the L1 protein [27, 37, 38, 39]. To identify which surface loops of HPV6 L1 are crucial for mAb recognition, we genetically substituted the surface loops or subloops of HPV6 L1 with corresponding ones of HPV16 L1 (BC, DEa, DEb, DEc, EF, FGa, FGb, FGc, HIa, HIb), and generated 10 chimeric HPV6-16 VLPs: H6-16BC, H6-16DEa, H6-16DEb, H6-16DEc, H6-16EF, H6-16FGa, H6-16FGb, H6-16FGc, H6-16HIa, H6-16HIb (Figure 1C and Figure S1A). Of note, loop-swapping for the DE, FG, and HI loops, which have relatively long sequences, was undertaken using subloops instead of the full sequence (DEa, DEb, DEc, FGa, FGb, FGc, HIa, HIb) to obviate any potential dramatic conformational changes that could occur upon long loop replacement. All chimeric VLPs assumed good self-assembled morphologies and comparable size with respect to the wild-type HPV16 and HPV6 VLPs in negative-staining TEM (Figure S1B). The potential binding regions for the mAbs against the HPV6 capsid were determined by variations in the EC_50_ values between the WT and loop-swapped chimeras in an ELISA assay (Table S2). We then plotted a heat map using the EC_50_ changes to illustrate the differences in the binding region profiles of the mAb; a threshold of >5-times indicates a substantially important effect of the loop/subloop swap (Figure 1D). The heat maps showed binding at the following regions of each mAb: 2D4 shows binding at the BC, DE, EF, and FG loops; 18E4 at least at EF and FG loops; 10H1 at BC, DE, FG, and HI loops; 17D5 at BC, DE, and FG loops; 11B10 at BC and FG loops; 5D3 at the FG loop; and 15F7 at DE and EF loops. Intriguingly, among the different antibodies, all five surface loops (BC, DE, EF, FG, and HI) of HPV6 L1 contributed to antibody recognition, with loops BC, DE, and FG predominantly associated with nAb binding. Importantly, the FG loop has critical sites for the binding of potent mAbs, as indicated by the loss of reactivity for six of the seven mAbs in the FG-swapped HPV6 VLPs. This loop profile appears to reflect the results from other studies of the well-characterized HPV16 and HPV59 [17, 38].

### Neutralization mechanism of HPV6 nAbs

HPV infection is multifaceted and includes stages of host cell attachment, entry and intracellular trafficking. Neutralizing antibodies are believed to interfere with at least one of these stages [40]. To elucidate how nAbs perturb the route of HPV6 PsV infection, HPV6 PsV activity was traced in HaCaT cells by immunoflurescence microscopy. The PsV was bound to the ECM or cell surface in the presence of an nAb, a non-neutralizing mAbs, or bovine serum albumin (BSA, negative control), and the fluorescence of the attached PsV was quantified by averaging the pixel numbers in 10 randomized microscopy views. BSA values were used for normalization.

**Figure 2.**
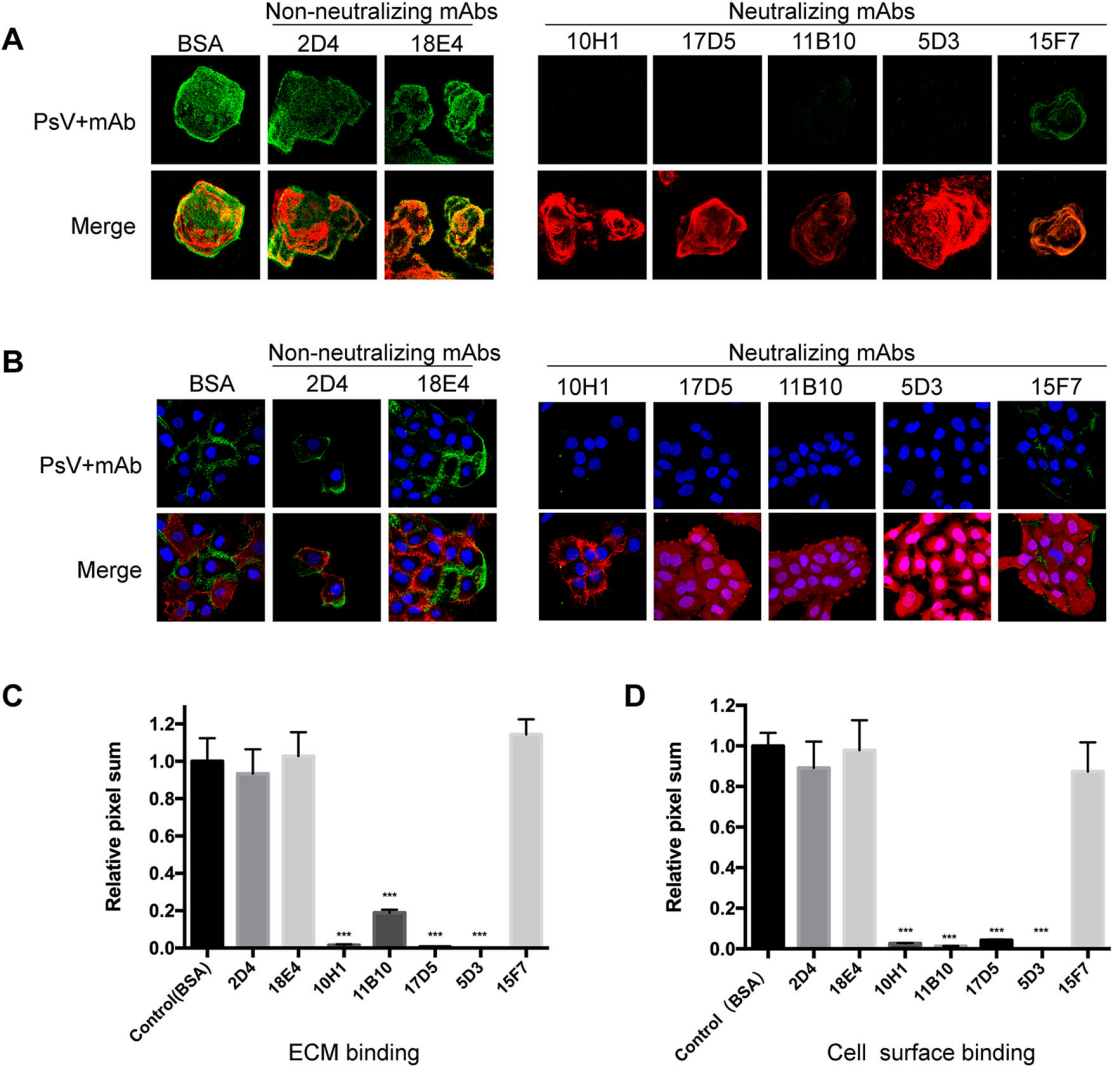
Attachment assay for HPV6 mAbs to the ECM and cell surface. HaCaT cells were prepared on coverslips for 48 h and then incubated with the HPV6 PsV premixed with each of the mAbs (five neutralizing mAbs and two non-neutralizing mAbs). Staining was performed with Alexa Fluor 488 (green). The PsV incubated with BSA served as the negative control. DAPI (blue) was used to stain for cell nuclei, laminin-5 (red) for the ECM in the ECM-binding assay (A), and phalloidin (red) for activity in the cell-surface binding assay (B). For each mAb, the merged images are shown in the lower panel and the PsV (green) in the upper panel. All images were quantified via LSM software (Zen) and ImageJ. (C, D) The pixel sum for virus bound with antibody is shown relative to that for virus with BSA control. ***P < 0.0001.

For the ECM attachment test, non-neutralizing mAbs (2D4 and 18E4) had minimal effect on prohibiting PsV attachment, as evidenced by highly positive green staining of the PsV in the ECM (Figure 2A, upper left panel). This is compared with the merged images showing the superimposition of the ECM in red and the PsV in green (Figure 2A, lower left panel). There is a comparable amount of attached PsV in the presence of either the non-neutralizing mAbs or the BSA negative control (Figure 2C). By comparison, four nAbs (10H1, 17D5, 11B10, and 5D3) were able to almost completely inhibit HPV6 PsV attachment to the ECM. Intriguingly, nAb 15F7 did not perturb PsV attachment to the ECM, with values similar to the non-neutralizing mAbs (Figure 2A right panel and Figure 2C). Finally, the inhibitory profile for cell surface attachment (Figure 2B and D) was almost identical to that for ECM attachment.

### Cryo-EM structure reconstruction of HPV6 PsV

**Figure 3.**
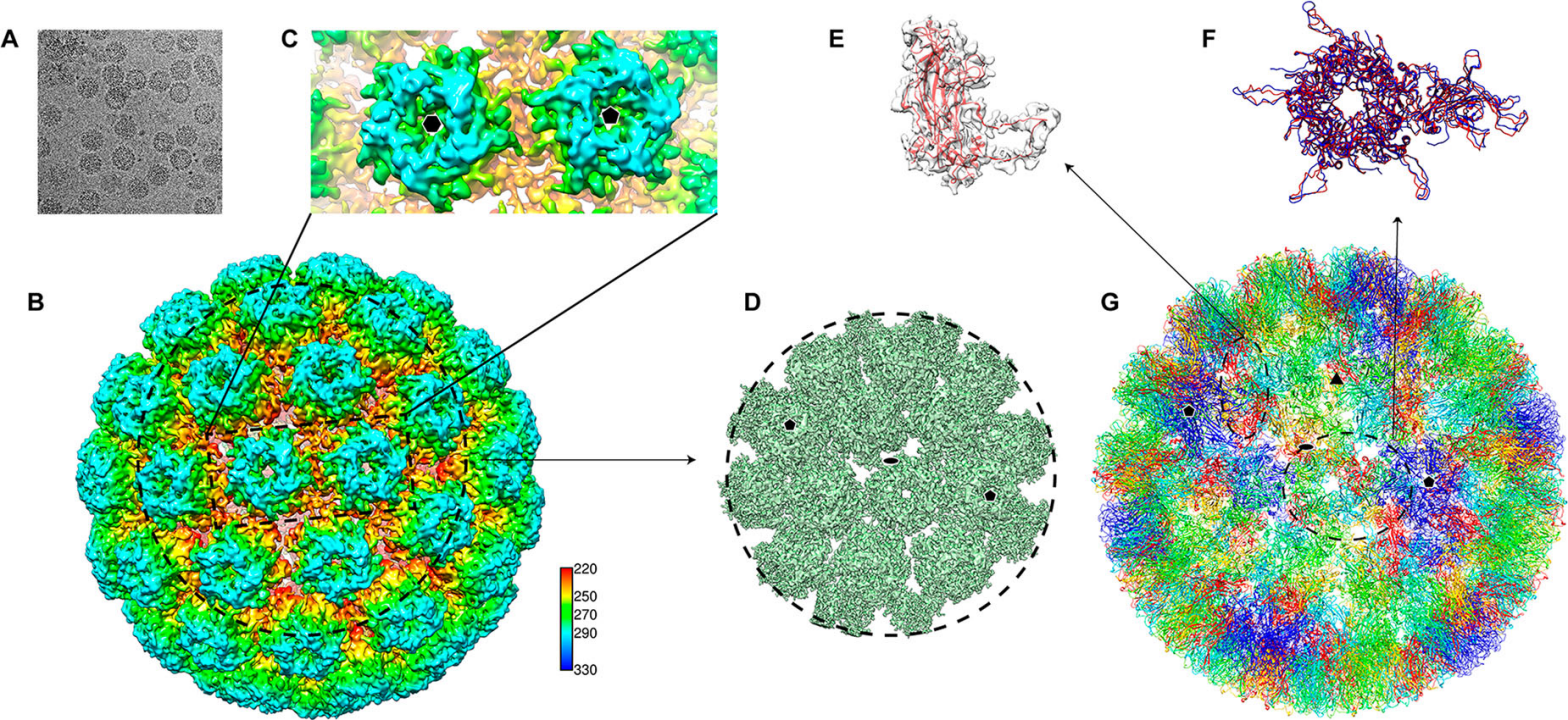
Cryo-EM reconstruction of HPV6 PsV at near-atomic resolution. (A) Representative cryo-EM images of the vitrified HPV6 PsV. (B) Density maps coloured according to the distance from the centre of the capsid: red, 220 Å; yellow, 250 Å; green, 270 Å; cyan, 290 Å; and blue, 330 Å. (C) Zoomed-in view of the HPV6 PsV includes a 5-coordinated pentamer (black pentagon) and a 6-coordinated pentamer (black hexagon). (D) Subparticle reconstruction of HPV6 PsV at the region of the 2-fold axes of icosahedral symmetry. (E) One HPV6 L1 monomer of the near-atomic model was fitted into the corresponding density map. (F) Structural comparison of the asymmetric units between the HPV6 (red) and HPV16 (blue) models. (G) Complete near-atomic model of HPV6 is shown in ribbon representation. Two-, three-, and five-fold axes of icosahedral symmetry are indicated as a black ellipse, triangle, and pentagon, respectively.

To identify the neutralization sites on the HPV6 capsid, we first prepared the HPV6 PsV alone according to our previous protocol [41] followed by vitrificaton and subsequent cryo-EM structure reconstruction. (This PsV preparation will later be used for various immune complexes with the aforementioned HPV6 nAbs.) The PsV displayed as homogeneous particles in the view of low-dose cryo-EM images (Figure 3A). We finally resolved the structure at a resolution of 5.5 Å, as determined by the gold-standard Fourier shell correlation (FSC), with a cutoff threshold of 0.143 (Figure S3, Table S1). Like previous overall structures of HPV particles [16, 17, 27, 38, 42], the HPV6 capsid displayed a T = 7d icosahedral structure, with a diameter of 58 nm, and the capsomers exhibited a pronounced star-shaped morphology (Figure 3B and C). The pentamer of HPV6 was easily identifiable, and “suspended bridges” (comprising the C-terminal arm of L1 proteins) connecting neighboring pentamers were conspicuous in the density map (Figure 3C).

The structure of the HPV6 PsV was further improved to 4.18 Å resolution by subparticle refinement (Figure 3D, S3); this allowed us to build a near-atomic model of the T = 7 HPV6 PsV structure, using the crystal structure of the HPV16 capsid (PDB ID:5KEP) as a starting model. The built model was refined with Phenix [35] (Figure 3G). In the structure, the HPV6L1 monomer exihibited well-resolved α-helix and β-sheet core regions and interpretable loop regions (Figure 3E). Overall, the asymmetric unit of the HPV6 model had a very similar core region to that of HPV16, with a root mean square deviation [RMSD] of 1.95 Å for all C-α atoms of L1. However, there were prominent differences in the surface loops and suspended bridges in the structural alignment (Figure 3F), believed to be associated with genotype specificity [41, 42].

### Cryo-EM reconstruction of HPV6 PsV in complex with nAbs

To localize the neutralization sites on the HPV6 capsid, we next determined the structures of the HPV6 PsV complexed with different nAbs by cryo-EM. Five potent nAbs (5D3, 17D5, 15F7, 11B10, 10H1) were used in the preparation of the immune complexes. For unknown reasons, Fabs 10H1 and 11B10 were not discernible, either in low-dose cryo-EM images or after 2D classification of selected particles (Figure S2). Thus, we only obtained structures for three immune complexes, HPV6:17D5, HPV6:5D3, and HPV6:15F7 at resolutions of 11.94, 16.55, and 4.36 Å, respectivley (FSC cutoff of 0.143; Figure S3, Table S1).

**Figure 4.**
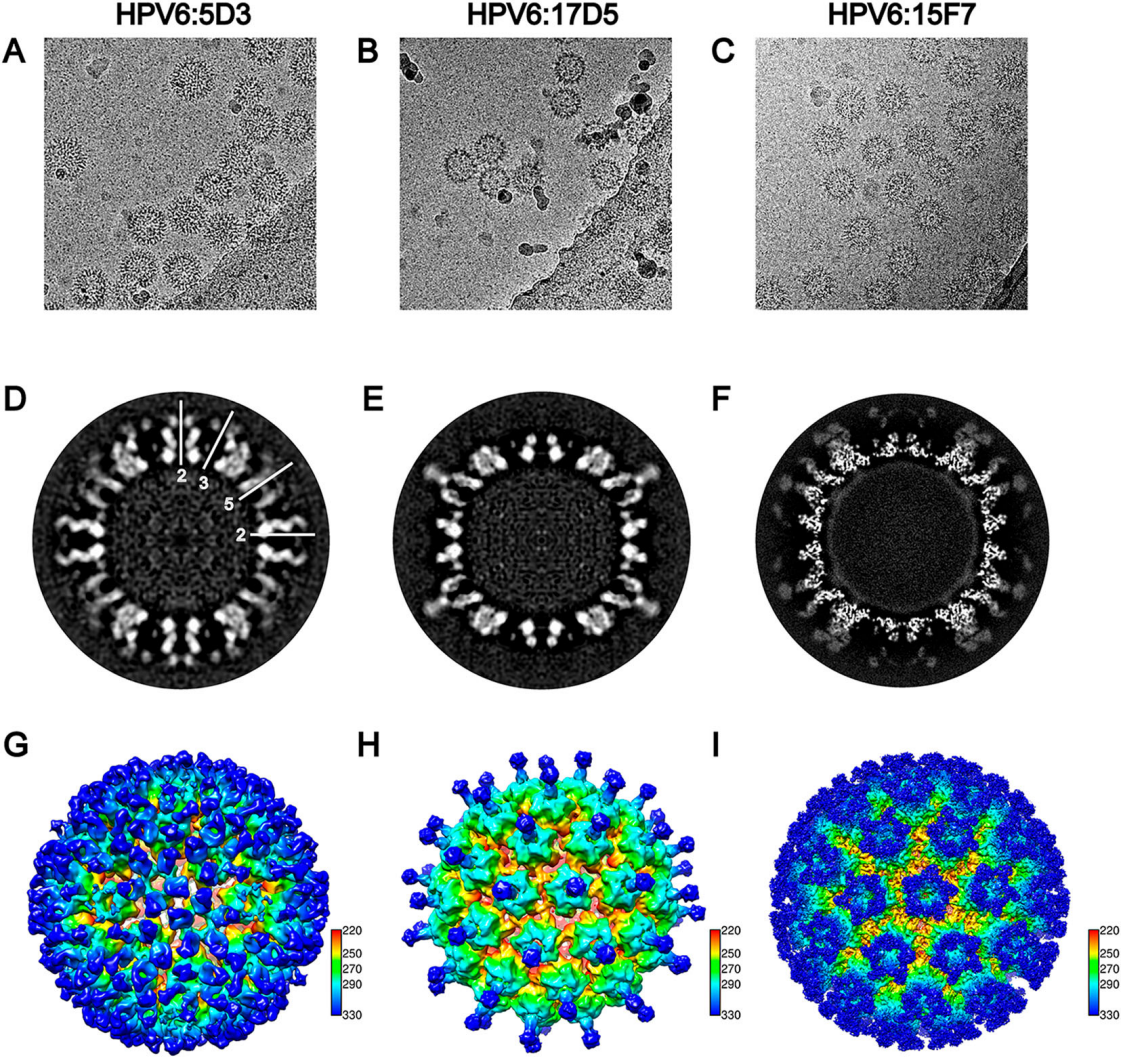
Cryo-EM reconstruction of the HPV6-Fab complexes. Representative cryo-EM images of the HPV6 PsV complexed with 5D3 (A), 17D5 (B), and 15F7 (C). (D, E, F) Central slice of the PsV-Fab density maps shown along icosahedral 2-fold, 3-fold, and 5-fold axes, respectively (white lines). (G, H, I) Density maps of complexes coloured according to the distance from the centre of the capsid.

Interestingly, we observed three different modalities of Fab binding in the cryo-EM reconstructions of the three immune complexes. In the case of Fab 5D3 binding, five Fabs engaged with each capsomer, with a total of 360 Fabs decorating the entire capsid in a full-occupation manner. However, the densities ascribable to the Fabs bound to the 5-coordinated pentamers were weaker than those of the 6-coordinated Fabs, which might be due to steric clashing of the five Fabs residing in the relatively smaller outside space of the 5-coordinated pentamers compared with the 6-coordinated ones (Figure 4A, D, G); this situation was also observed in the reported cryo-EM structure of the immune complex HPV16.V5 in a previous study [17]. In contrast, a single Fab 17D5 binds to the centre of the HPV6 pentamer, with a nearly vertical orientation against the pentamer surface, a so-called top-centre binding mode (Figure 4B, E and H); we previously reported a similar binding for the crystal structure and cryo-EM structure of the HPV58.A12A3 complex [38]. Finally, unlike previously resolved HPV capsid–antibody binding modalities, we observed a unique mode of binding for 157F. We show that five 15F7 Fabs cluster intensively around each pentamer (top-rim binding mode) with 360 Fabs decorating the capsid as 72 five-spade petals (Figure 4C, F and I). Thus, 15F7 binding may reflect a new mode of antibody binding in the neutralization of the HPV capsid.

**Figure 5.**
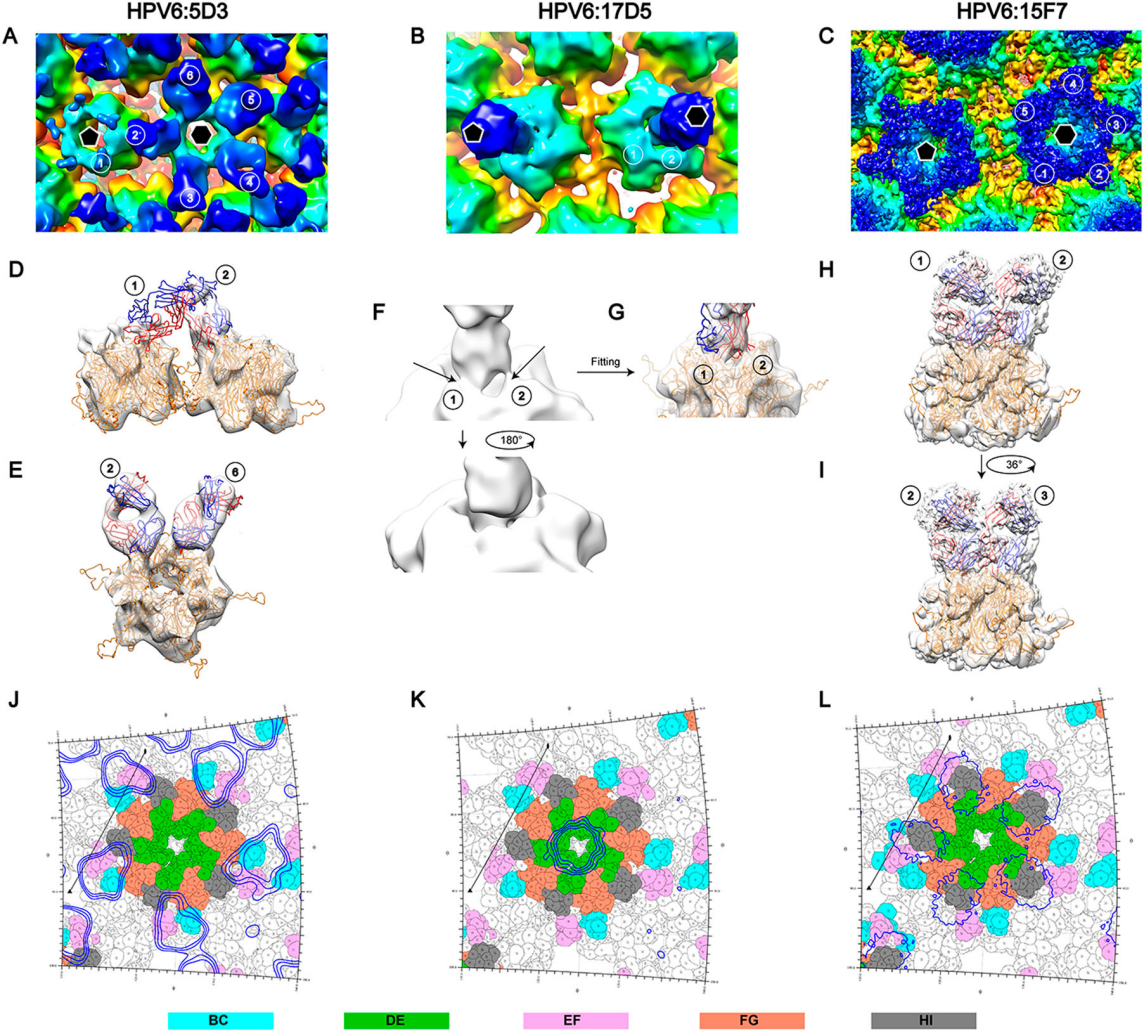
Binding interface and footprint of HPV6 nAbs. An asymmetric unit of the HPV6 PsV structure and crystal structure of a murine antibody (PDB accession code 3RKD) were fitted to the cryo-EM density map (gray mesh). Light and heavy chains of the Fab and the HPV6 L1 protein are coloured in blue, red and orange, respectively. (A, B, C) Zoomed-in view shows a pentavalent and the neighboring hexavalent capsomers, as indicated by the pentagon and hexagons, respectively. (A) The numbers denote the six different monomers of the icosahedral asymmetric unit on the map of the HPV6:5D3 complex. (D, E) A map of a pentamer with different Fabs was extracted and fitted using the structure of the HPV6 particle and crystal structure of Fab. (B) The numbers denote two different monomers of the pentavalent capsomer on the map of the HPV6:17D5 complex. (F) Close-up view of interface between the Fab and capsomer. (G) Density map was superimposed with the above-mentioned crystal structure. (C) The numbers denote five different monomers of the pentavalent capsomer on the map of the HPV6:15F7 complex. (H, I) The same density map was superimposed with the above-mentioned crystal structures. A stereographic projection was used to show the surface of the particles, where the polar angles θ and Φ represent latitude and longitude, respectively. (J, K, L) Footprints of three Fabs (5D3,17D5,15F7) displayed by deep blue contour lines. The BC, DE, EF, FG, and HI surface loops of the HPV6 L1 protein are shown in cyan, green, purple, salmon, and gray, respectively. The locations of the 2-fold and 3-fold icosahedral symmetry axes are indicated as black ovals and triangles.

Next, the aforementioned model of the HPV6 PsV and the crystal stucture of a murine monoclonal antibody Fab (PDB: 3RKD) were fitted into cryo-EM electronic density maps of the immune complexes for further epitope analysis. Similar to the cryo-EM structure of HPV16:V5 and HPV58:28F10 complexes [16, 38], six different 5D3 Fabs – located on six monomers in the asymmetric unit of the capsid – exhibited varied occupancies in the density map of HPV6:5D3 (Figure 5A). Upon rigid fitting of HPV6, a steric clash was observed between Fab-1 from the 5-coordinated pentamer and Fab-2 from the 6-coordinated pentamer (Figure 5D). However, adjacent Fabs within one pentamer, e.g. Fab-2 and Fab-6, showed no steric interference with each other (Figure 5E). It is reasonable to propose that 5D3 can freely bind to locations 3, 4, 5, and 6 and prefer to attach to location 2 rather than location 1 because of a stronger density for Fab-2 than for Fab-1.

In the case of HPV6:17D5, we found two combined densities linking the Fab with monomer 1 and 2 in the 6-coordinated pentamer (Figure 5B and F). According to the fitting result (Figure 5G), we infer that 17D5 can attach to the HPV6 capsid in a specific orientation. Unlike 5D3, 15F7 Fab clustering around each pentamer (including 5-coordinated and 6-coordinated ones) is distant and free from any interaction with other Fabs bound to neighboring pentamers (Figure 5C). In addition, the five Fabs might associate with each neighboring other within one cluster, as noted by their close positions in both the cryo-EM density map and the fitted model (Figure 5H and I); the 5-fold association of five capsid-bound Fabs has been observed previously in the atomic structure of the coxsackievirus A6 immune complex [43]. In addition, the variable domains of the bound 15F7 Fab have a stronger density than those of the other two Fabs, which suggests full occupation of the binding sites of 15A7 Fab on the HPV6 capsid.

The potential bivalent binding of full-length antibody for all the three mAbs was analyzed by modeling the full-length antibody and measuring the distance between two most closely adjacent Fabs bound to the capsid. Interestingly, it is possible for mAbs 5D3 and 15F7 to bivalently bind to a single particle. However, both Fabs of one full-length antibody 17D5 cannot simultaneously bind to a single capsid due to long distance (ranging 154–169 Å) between two neighboring bound Fabs (Figure S4). In addition, negative staining TEM assay of VLP:nAbs complexes confirmed the neutralizing antibodies could bind to HPV6 capsid in full-length form as well as their Fabs did in cryo-EM. Intriguingly, non-neutralizing antibodies 2D4 and 18E4 could not bind HPV6 VLPs (Figure S5), which is inconsistent with that they were well reactive in VLP-based ELISA binding assay (Figure 1B). On the other hand, they were identified to recognize linear epitopes in WB test (Table S3). Taken together, 2D4 and 18E4 might recognize some linear sites not exposed in solution-state particle but accessible to antibody binding in case of VLPs coated in the microplate.

### Epitope mapping of three HPV6 nAbs

The footprints of 5D3, 17D5, and 15F7 on the HPV6 capsid were delineated by projecting the density map onto the surface of the model of the HPV6 particle using RIVEM software [44]. According to these footprints, the binding sites of Fab 5D3 cover five conformationally close regions: K52, R53, A54, and N55 of the BC loop; K169, T172, N173, P175, V176, Q177, and A178 of the EF loop; E262, V263, E265, and P266 of the FGa loop; V344, T345, and T346 of the HIa loop; and S353 of the HIb loop (Figure 5J). Among these regions, the FG loop plays a critical role in the interaction between 5D3 and the HPV6 capsid, as indicated in the binding assay using loop-swapped HPV6 VLPs (Figure 1D, Table S2). For the binding of 17D5 Fab, the DE loop (DEa: Y123, N128; DEb: G130, S131, G132; DEc: N139) and the FG loop (N278 and R279) are involved in the capsid-antibody interactions (Figure 5K); this is consistent with the results of the binding variations with loop-substituted HPV6 VLPs, where the substitution of DEa, DEc, or the FGb loops of HPV6 with those of HPV16 abrogates the binding activity of mAb 17D5 with the VLPs (Figure 1D, Table S2). As compared with the other two antibodies, the binding regions of 15F7 span a greater number of surface loops, including the DEb loop (N134, P135, G136, Q137), DEc loop (N139), EF loop (K169, V176, Q177, A178), FGa loop (E262, V263), FGb loop (S276, G277, N278), HIa loop (V344, T345, T346) and HIb loop (S347, S348, T349, S353) (Figure 5L). These interaction sites overlap with regions clustered in the DEb, DEc, and EF loops, as revealed in binding tests (Figure 1D, Table S2).

## Discussion

Currently, most of the information regarding virus neutralization centres around high-risk HPV types. For instance, the classical neutralizing antibody, HPV16.V5, has been extensively characterized through structural analysis and immune assays for its neutralization efficacy of the high-risk HPV16, whereas there are few in-depth studies related to the conformational epitopes of HPV6, the most abundant causative pathogen for genital warts. A previous study identified a region spanning residues 49–54 on the L1 protein of HPV6 as a neutralization site, using a CRPV/HPV6 hybrid L1 protein [28]. However, further detailed information pertaining to the mode of neutralization and how antibodies would be useful against this type of HPV are lacking. Here, we use a well-characterized anti-HPV6 monoclonal antibody panel [29] of several potent neutralizing antibodies (Table S3) to explore in detail the binding of neutralizing antibodies to HPV6.

Given the genotype-specific nature of most modes of HPV neutralization, we employed chimeric HPV6-16 VLPs bearing individual surface loop/subloop swapping to roughly map the neutralization sites of HPV6. This mode of analysis afforded the use of high-throughput ELISA followed by cryo-EM reconstruction to precisely map the neutralization sites. However, for HPV6:17D5 and HPV6:5D3, the binding modality – in particular, the overlap of neighboring Fabs – while allowing for binding to the capsid simultaneously, led to a low binding affinity and potential capsid structure perturbance that mitigated resolving a high-resolution structure. By comparison, HPV6:15F7 resulted in a high-resolution structure (Table S1) at least in part due to the favourable binding of all Fabs without any steric hindrance. Overall, we acquired three different binding modes for the HPV6 nAbs – dispersive capsid bound, top-centre-bound, and top-rim pentamer-bound – and obtained precise neutralization sites using the RIVEM method [44]. Moreover, our results describe an unprecedented manner of binding for nAb 15F7, as shown by the intensive clustering around every pentamer. It should be mentioned that a previously described [16] crystal structure of HPV16L1 bound with HSPG – deemed as a primary receptor for numerous viruses [45] – suggested that the region circumscribed by K54, K59, K278, K356, K361, K442, and K443 was associated with viral neutralization when using monoclonal antibodies for which the footprint befalls or overlaps with this region. Indeed, in the present study, we show that nAb 5D3 appears to neutralize the virus by interfering with HSPG attachment.

**Figure 6.**
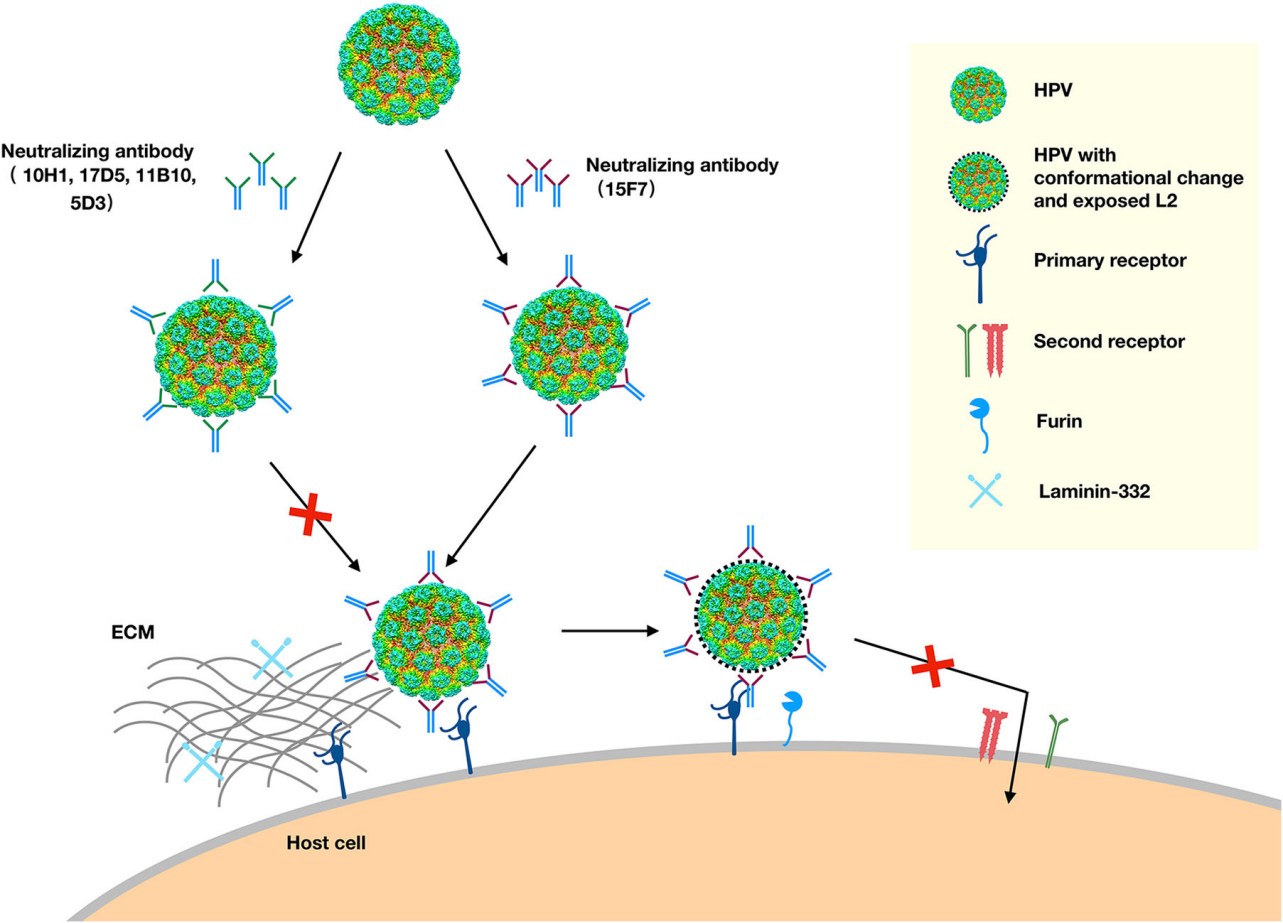
Proposed neutralization mechanism for mAbs against HPV6. HPV can attach to the host cell by binding to the primary receptor, such as heparan sulfate proteoglycans (HSPGs) on the cell surface or laminin-332 in the ECM. The virus then likely undergoes some conformational changes that expose the N-terminal amino acids of the minor L2 protein. Following furin cleavage of L2, the virions transfer to secondary receptors and initiate cell entry. Neutralizing antibodies (nAbs) recognize the surface epitopes of HPV. Some nAbs, like 10H1, 17D5, 11B10, and 5D3, block viral primary attachment to the ECM or cell surface. Others, such as 15F7, do not interfere with cell binding but may neutralize HPV by interrupting viral transfer on the cell surface or by altering the actions of secondary receptors responsible for internalization.

Viral neutralization is essential for organisms to fight against various pathogens in vivo. Neutralization mechanisms are usually related to antibody efficacy and action space as a function of time in the host, and are categorized pursuant to the perturbance, thus pointing to the influence of different phases of virus infection. Non-enveloped HPV infection is believed to involve host cell attachment, entry and intracellular trafficking, with the L1 major protein mediating attachment to cell receptors, facilitating exposure of the L2 protein to furin, and enabling its subsequent enzymatic cleavage, and finally, enabling the structural rearrangement that triggers the entry process (Figure 6) [24]. In this study, we show that nAb 5D3 recognizes a wide region, covering loops BC, EF, FG, and HI, which overlaps with the binding sites of HSPG, thereby encumbering viral attachment to both the ECM and the cell surface. Thus, it is reasonable to infer that 5D3 neutralizes HPV infection by blocking virus attachment and any sequential conformational changes that drive further viral internalization. Unlike 5D3, a single 17D5 moiety engages with the centre of the pentamer in a top-centered binding mode, as described previously by us for nAb HPV58.A12A3 [41]. The antibody footprints by this mode of binding mainly situate a region of the DE loop that is distant from the well-defined HSPG binding sites [23]. However, unexpectedly, 17D5 both in full-length and Fab forms could still prevent HPV6 PsVs from attaching to cell surface, which suggests that 17D5’s binding might induce conformation changes on or near HSPG binding sites in its neutralization mechanism (Figure 2B, Figure S6). Interestingly, the third nAb we characterized in detail, 15F7, strongly binds around each pentamer to confer potent neutralizing efficacy in the neutralization assay but still allows the virus to attach to the ECM and the cell surface (Figure 1B). Thus, 15F7 may neutralize the virus in post-attachment events (Figure 6) same as HPV16.V5 antibody, although HPV16.V5 can block viral attachment to ECM but allow engagement to cellular surface [26].

Overall, we comprehensively characterized and structurally mapped the major neutralization epitopes of HPV6, our results suggest two neutralization mechanisms for HPV6 nAbs: most nAbs neutralize HPV6 by interfering with the attachment phase of virus infection, whereas some nAbs (e.g. 15F7) affect virus penetration and entry into the host cell; albeit, in such cases, the Ab-bound virus still attaches to the ECM and/or the cell surface (Figure 6). It should be noted that the post-attachment neutralization of nAb 15F7 – a unique cluster-binding around each pentamer that allows cell receptor to bind to the unoccupied regions of the virus, but abrogates the Ab-bound virus from cell entry – might define a potential alternative mode of classic HPV inhibition by the attachment blocking of a non-HSPG cell receptor. These findings will expand our knowledge on HPV immunology and virology for the benefit of vaccine and antivirus design.

## Supplementary Material

Supplemental Material
