# Supplementary material for: Neutralization sites of human papillomavirus-6 relate to virus attachment and entry phase in viral infection
Source: Emerg Microbes Infect. 2019 Nov 26;8(1):1721–33. doi: 10.1080/22221751.2019.1694396 (PMC6883418; doi:10.1080/22221751.2019.1694396)
Supplement: Supplemental Material [file TEMI_A_1694396_SM2754.docx]

**Supplementary Information**

**
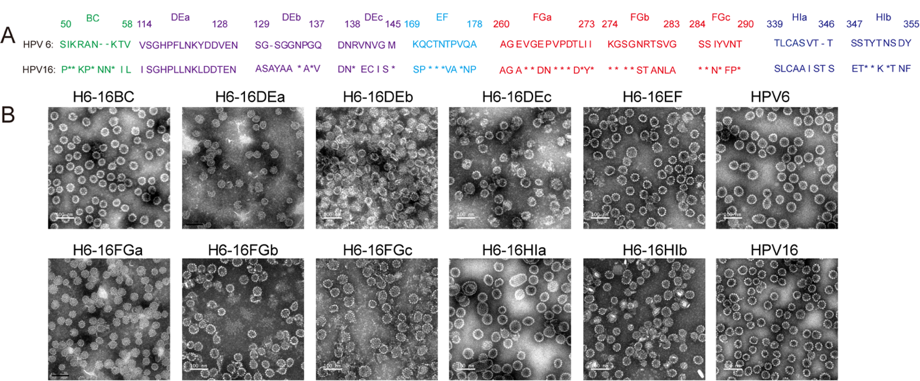
**

**Figure S1: Construction of HPV6-16 loop swapping chimeric virus-like particles (VLPs).** (A) Sequences of different surface loops from HPV6 (the upper panel) and HPV16 L1 (the lower panel) protein: green (BC loop), purple (DEa, DEb, DEc), blue (EF), red (FGa, FGb, FGc), deep blue (HIa, HIb). (B) TEM images of chimeric VLPs displayed a similar morphology as compared with wild-type HPV particles (final column).


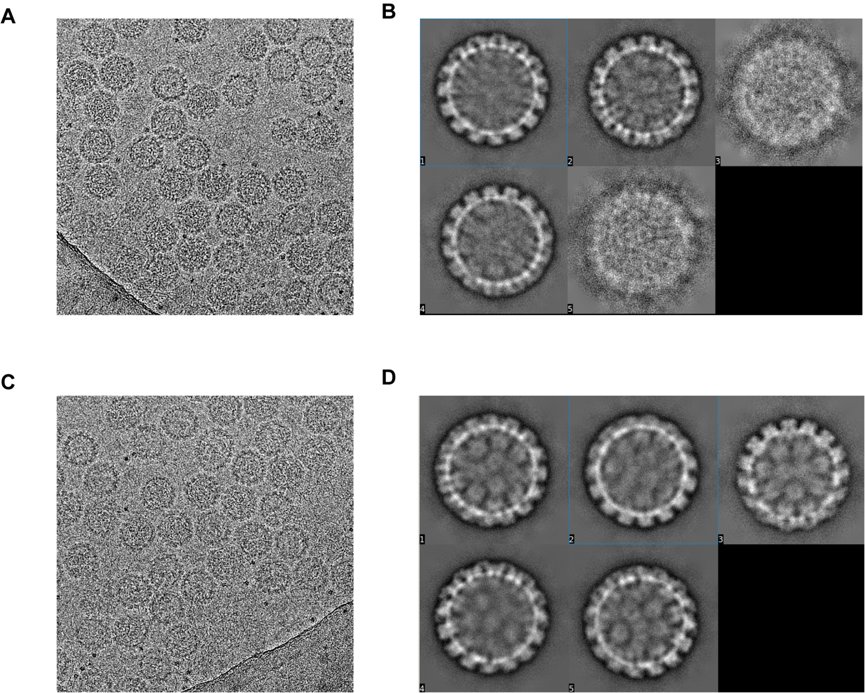


**Figure S2: Image and 2D classification of the immune complexes.** There is no visible density of Fab on HPV6:11B10 (A, B) and HPV6:10H1 (C, D) complexes.


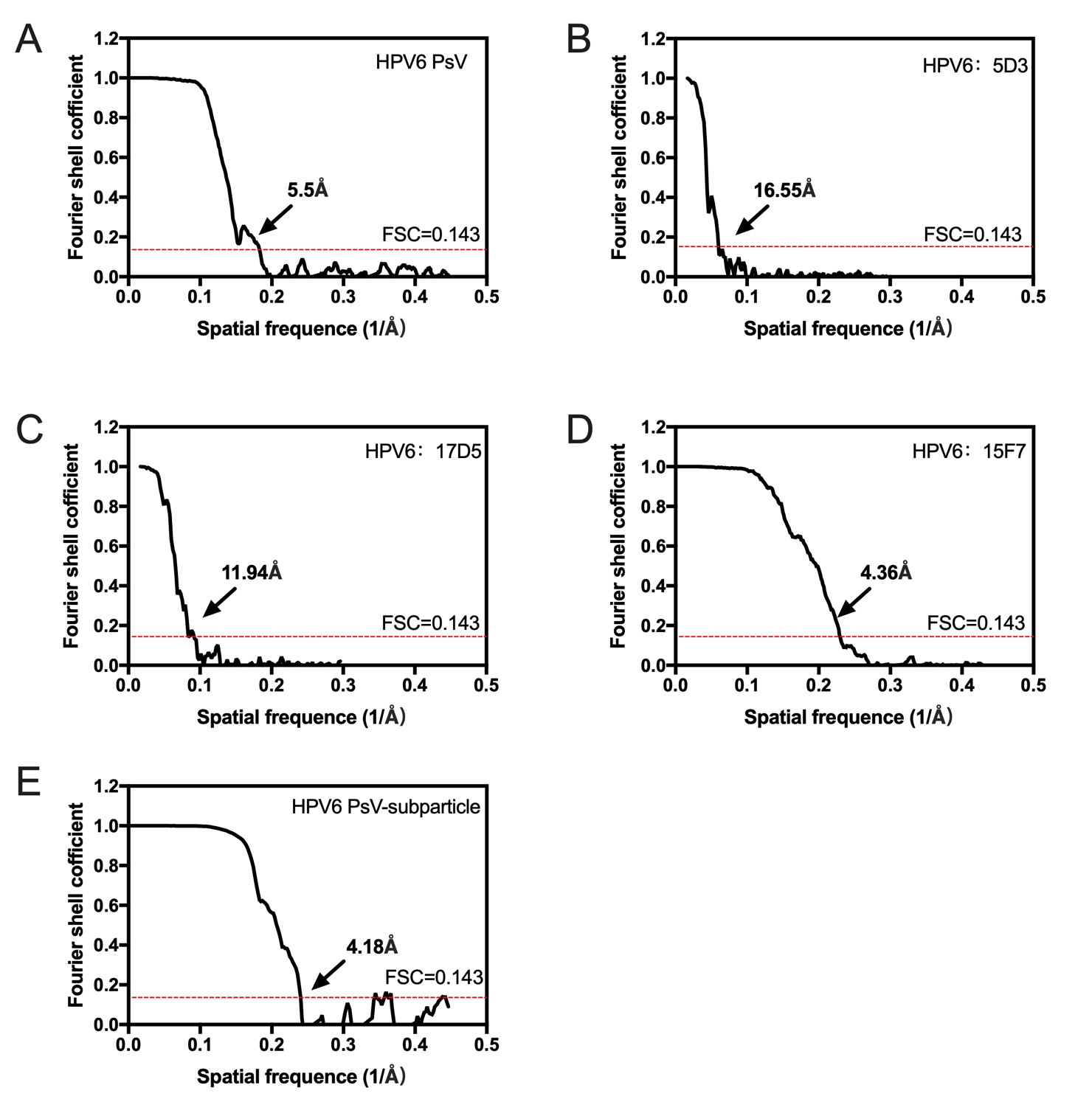


**Figure S3: Gold-standard Fourier shell correlation (FSC) curves of the density maps of HPV6 particles and HPV6-Fab complexes.**

**
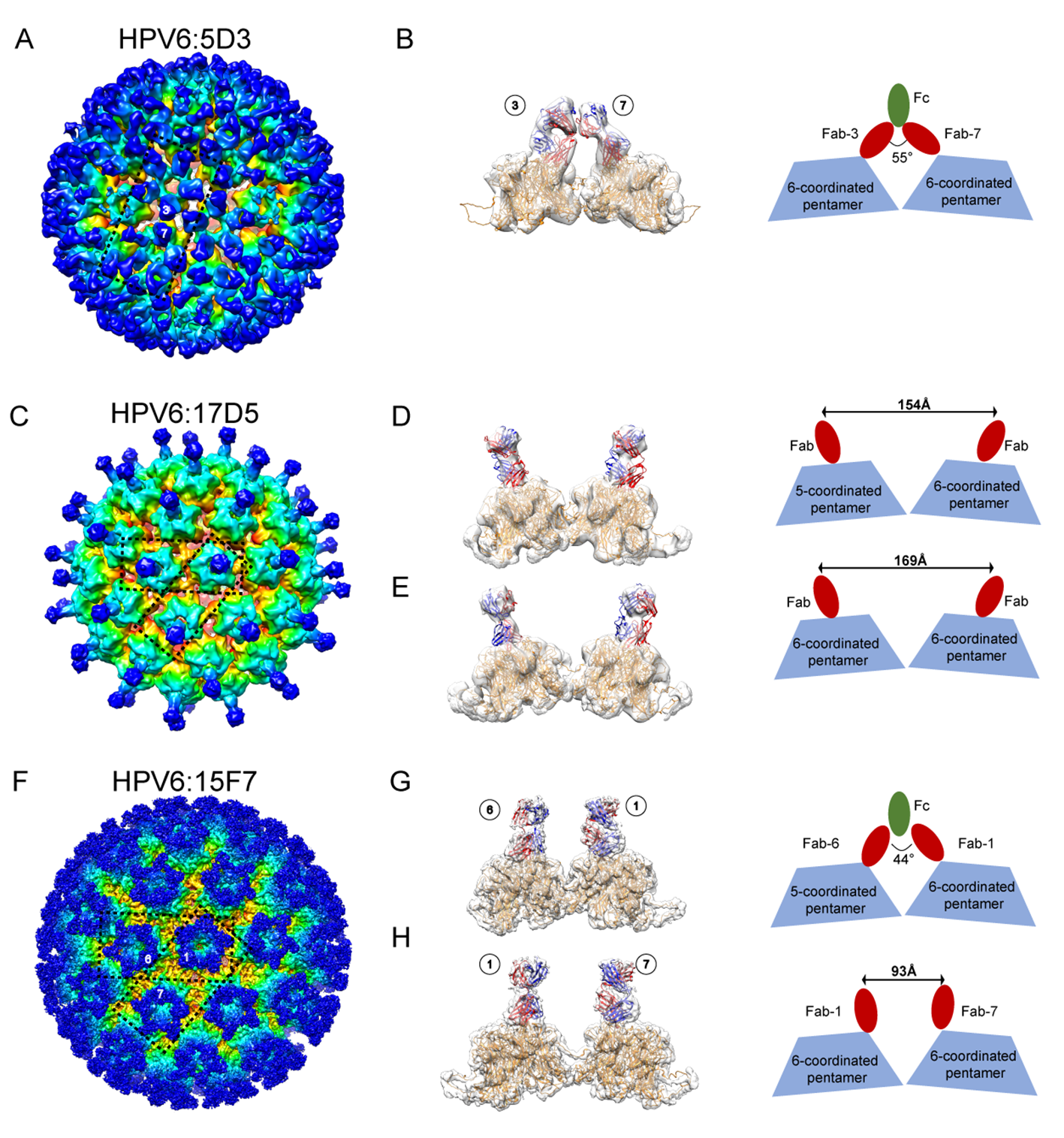
**

**Figure S4: Prediction of bivalent binding potential for full-length antibodies.** Overall density maps of HPV6:5D3 (A), HPV6:17D5 (C) and HPV6:15F7 (F). Two adjacent pentamers representing unique neighboring Fabs binding astride two pentamers were boxed by black dotted rectangles, and the density knobs of corresponding pair of pentamers in complex with two neighboring Fabs were extracted and fitted by the structure of two HPV6 pentamers and the crystal structure of one Fab (PDB ID:3RKD) to check the bivalent binding potential of 5D3 (B), 17D5 (D, E), 15F7 (G, H). The schematic representations for one full-length antibody binding to neighboring pentamers or distance between two separate Fabs that could not belong to single full-length antibody were shown in the right panels.

**
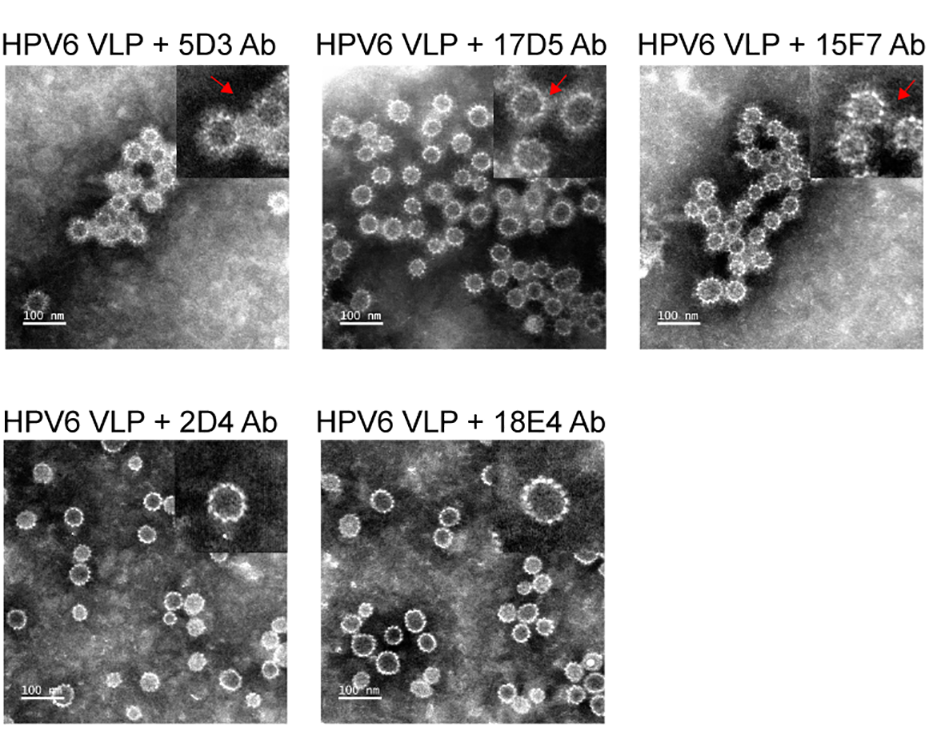
**

**Figure S5: Negative staining TEM of HPV6 VLPs incubated with 5 full-length antibodies.** The red arrows indicate the neutralizing antibodies 5D3, 17D5 and 15F7 can bind to HPV6 VLPs and even cross-bind two VLPs. However, no non-neutralizing antibodies 2D4 and 18E4 could be discerned to bind HPV6 VLPs.

**
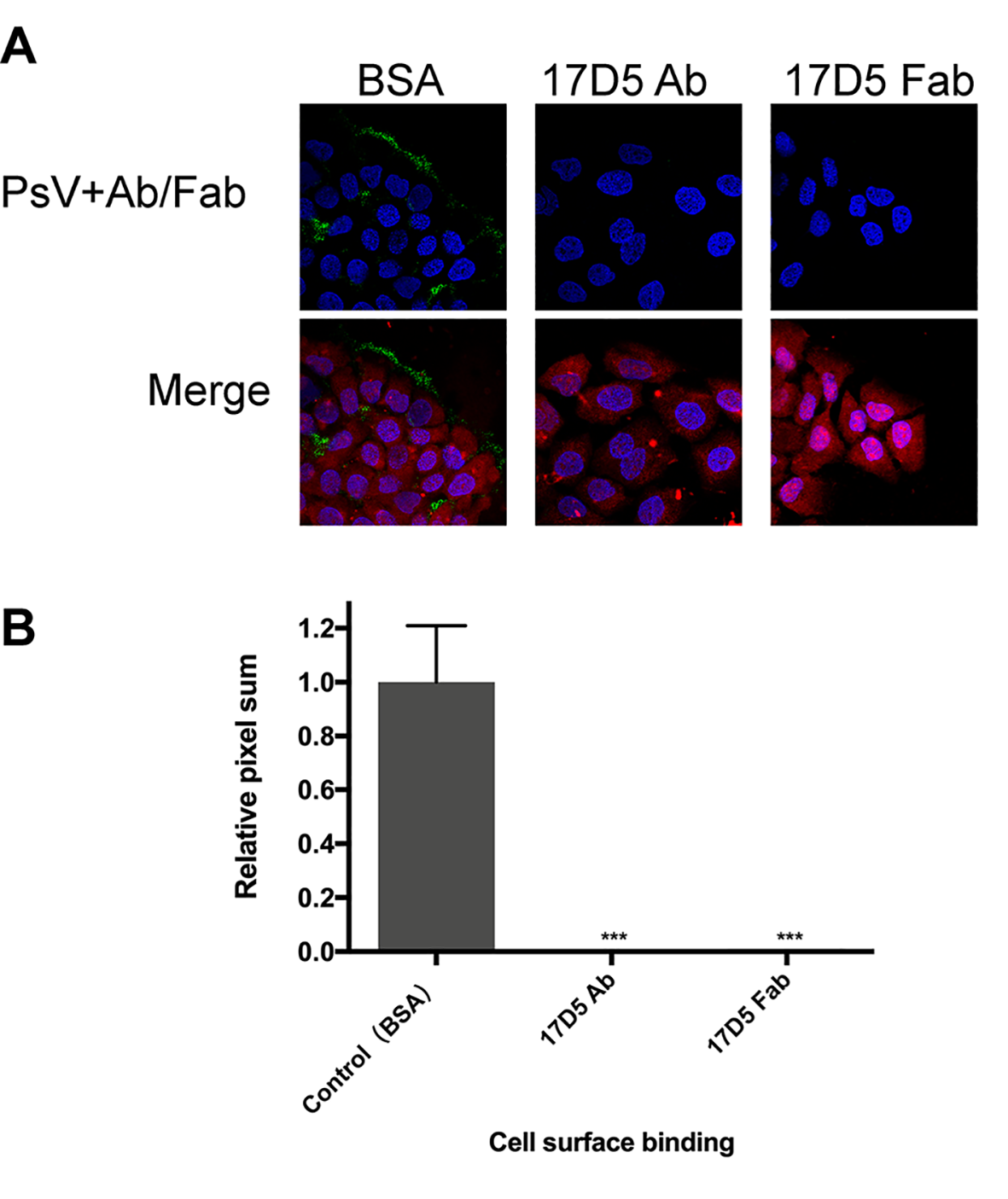
**

**Figure S6: Attachment assay of HPV6 PsVs to the cell surface in the presence of 17D5 or its Fab.** HaCaT cells were incubated with the HPV6 PsVs treated with 17D5 IgG or Fab (A). The PsVs incubated with BSA served as the negative control. The pixel sum for virus bound with antibody is shown relative to that for virus with BSA control. ***P < 0.0001.

**Table S1：Cryo-EM data collection and reconstruction**

| Samples | HPV6:5D3 complex | HPV6:17D5 complex | HPV6:15F7 complex | HPV6 PsV | HPV6 PsV-subparticle |
| --- | --- | --- | --- | --- | --- |
| Data collection | | | | |  |
| EM equipment | Tecnai F30 | | | | |
| Voltage (kV) | 300 | | | | |
| Detector camera | Falcon II | | Falcon III | | |
| Pixel size (Å) | 1.128 | | 1.120 | | |
| Electron dose (e^-^/ Å^2^) | 25 | | 30 | | |
| Frames | 17 | | 39 | | |
| Defocus range(µm) | 0.45-4.38 | 0.55-4.61 | 0.16-3.79 | 0.28-4.11 | 0.28-4.11 |
| Reconstruction | | | | |  |
| Software | AUTO3DEM/RELION | | cisTEM | |  |
| Particle number | 1367 | 1201 | 5569 | 5783 | 173490 |
| Final Resolution (Å) | 16.55 | 11.94 | 4.36 | 5.5 | 4.18 |

| mAb | HPV6 VLP | HPV6-16BC | HPV6-16DEa | HPV6-16DEb | HPV6-16DEc | HPV6-16EF | HPV6-16FGa | HPV6-16FGb | HPV6-16FGc | HPV6-16HIa | HPV6-16HIb |
| --- | --- | --- | --- | --- | --- | --- | --- | --- | --- | --- | --- |
| 2D4 | 122.50* | 1165.00 | 1466.00 | 169.00 | 23.60 | 1640.00 | 66.66 | 56.72 | >3000 | 75.88 | 462.20 |
| 18E4 | 180.50 | 407.20 | 593.20 | 244.00 | 168.40 | 1899.00 | 92.96 | 228.50 | 1910.00 | 55.62 | 86.95 |
| 10H1 | 7.57 | 583.40 | >3000 | 42.06 | 27.70 | 17.52 | 71.46 | 314.80 | 25.33 | 50.38 | 40.33 |
| 17D5 | 39.12 | 298.20 | >3000 | 103.50 | 495.80 | 49.82 | 28.15 | 317.30 | 29.53 | 22.21 | 57.45 |
| 11B10 | 13.29 | >3000 | 25.41 | 22.95 | 24.64 | 28.57 | >3000 | 2342.00 | 23.51 | 9.78 | 11.02 |
| 5D3 | 45.68 | 72.50 | 52.04 | 177.30 | 55.76 | 34.14 | 43.55 | >3000 | 56.80 | 28.21 | 199.80 |
| 15F7 | 118.70 | 460.40 | >3000 | >3000 | 925.30 | >3000 | 228.10 | 562.10 | 221.90 | 205.10 | 444.60 |

**Table S2: Quantitative analysis of anti-HPV6 antibodies binding to chimeric VLPs of HPV6-16 via ELISA.**

* The value denotes EC_50_ (ng/ml) calculated by the curve plotted OD_450nm_ vs. input antibody concentrations using software Prism7.

**Table S3: Characteristics of murine anti-HPV6 monoclonal antibodies.**

| mAb ^a^ | Epitope type | Isotype | Binding affinity  EC_50_（ng/ml） | Neutralizing affinity  IC_50_（ng/ml） | Inhibition mechanism ^b^ | |
| --- | --- | --- | --- | --- | --- | --- |
|  |  |  |  |  | ECM | cell surface |
| Ⅰ Non-neutralizing antibody | | | | | | |
| 2D4 | L | IgG2a | 122.5 | >1000 | + | + |
| 18E4 | L | IgG1 | 180.5 | >1000 | + | + |
| Ⅱ Neutralizing antibody | | | | | | |
| 10H1 | C | IgG1 | 7.57 | 0.76 | - | - |
| 17D5 | C | IgG2a | 39.12 | 0.94 | - | - |
| 11B10 | C | IgG1 | 13.29 | 1.63 | - | - |
| 5D3 | C | IgG2b | 72.5 | 2.97 | - | - |
| 15F7 | C | IgG1 | 118.7 | 8.81 | + | + |

^a^ mAbs were classified into two groups according to the neutralizing efficiency.

^b^ “+” and “-” indicate the virus incubated with the relative mAb that could bind to the ECM, cell surface or neither.
